# High levels of retinal docosahexaenoic acid do not protect photoreceptor degeneration in *VPP* transgenic mice

**Published:** 2010-08-18

**Authors:** Feng Li, Lea D. Marchette, Richard S. Brush, Michael H. Elliott, Kimberly R. Davis, Ashley G. Anderson, Robert E. Anderson

**Affiliations:** 1 Department of Ophthalmology, University of Oklahoma Health Sciences Center, Oklahoma City, OK; 2 Department of Cell Biology, University of Oklahoma Health Sciences Center, Oklahoma City, OK; 3 Dean A. McGee Eye Institute, Oklahoma City, OK

## Abstract

**Purpose:**

To determine whether docosahexaenoic acid can protect against hereditary retinal degenerations in transgenic mice expressing the V20G, P23H, and P27L (VPP) rhodopsin mutations.

**Methods:**

Female transgenic mice expressing the VPP rhodopsin mutation, known to cause a retinal degeneration, were bred to male transgenic mice expressing the *fat-1* gene, which can convert n6 to n3 polyunsaturated fatty acids (PUFA). Several weeks before breeding, the female mice were fed a standard diet containing 10% safflower oil (SFO), which is high in n6 and low in n3 PUFA (n6/n3=273). Offspring were genotyped and four groups identified: Fat1^+^/VPP^+^, Fat1^–^/VPP^+^, Fat1^+^/VPP^–^, and Fat1^–^/VPP^–^. Dams were maintained on the SFO diet during the nursing period and offspring were kept on the SFO diet after weaning. At 4, 16, and 28 weeks of age, retinal function was evaluated by electroretinography (ERG), photoreceptor cell loss was quantified by measuring outer nuclear layer thickness, and rhodopsin levels were determined. Fatty acid profiles were analyzed in whole retina, plasma, and liver at 4 and 28 weeks of age.

**Results:**

Expression of *fat-1* in the absence of dietary n3 PUFA led to the generation of two groups of mice with distinctly different levels of n3 and n6 PUFA in the three tissues that were analyzed. Already at four weeks of age, the retinas of *fat-1* positive animals had higher levels of n3 PUFA than their wild-type counterparts (23%–29% versus 6.4%–6.5%). In addition, by four weeks of age, there was a significant loss of rod photoreceptor cells in the VPP mice. Progression of retinal degeneration occurred with increasing age in *VPP* positive mice, with photoreceptor cell death occurring in both inferior and superior regions. Amplitudes of the a- and b-waves of the ERG were significantly reduced with age, with *VPP* positive mice showing the greatest change. Rhodopsin content was lower in the VPP transgenic mice. There were no significant differences in outer nuclear layer thickness or ERG amplitudes between Fat1^+^/VPP^+^ and Fat1^–^/VPP^+^, or between Fat1^+^/VPP^–^and Fat1^–^/VPP^–^ mice at any of the three ages.

**Conclusions:**

High levels of retinal docosahexaenoic acid do not protect mice expressing the VPP rhodopsin mutation from retinal degeneration.

## Introduction

High levels of docosahexaenoic acid (DHA; 22:6n3), a member of the n3 family of essential polyunsaturated fatty acids (PUFA), are found in rod photoreceptor outer segments (ROS) [1]. Studies over several decades have shown that DHA is essential for maintaining the normal structure and function of the retina [2–9]. Reduction of DHA in the retina can lead to diminished retinal electrical responses to light in rodents [2,^3^,^10^], reduced visual acuity in nonhuman primates [4,5], and delayed photoreceptor development and function in preterm and term human infants [6,8].

A large number of studies have shown that the levels of DHA and other n3 and n6 PUFA are reduced in the blood of patients with retinitis pigmentosa (RP) compared to appropriate controls, regardless of the mode of inheritance [11–21], with the greatest differences occurring in patients with X-linked RP [18]. Animals with mutations causing retinal degenerations in humans also have lower levels of n3 and n6 PUFA in their blood [22–26]. In several animal models of RP, the level of DHA in retinas [26] and rod outer segments [25,27–29] is lower than that found in controls, suggesting that the reduced DHA may in some way contribute to or exacerbate the retinal degeneration in this group of animals. Indeed, a clinical trial involving humans given DHA found a subgroup of patients that responded positively to supplementation [30], although there was no beneficial effect for the majority of the patients in the study [30,31]. Studies in X-linked patients have also shown a minimal benefit of DHA supplementation, again in a sub-group of patients [32]. A previous study in dogs with progressive rod-cone degeneration found no benefit in the supplementation of fish oil [25], which contains large amounts of n3 PUFA. Likewise, supplementation of DHA in rats with P23H or S334ter rhodopsin mutations did not prevent or slow the loss of rod photoreceptors [33]. Finally, we recently showed that the level of DHA in the retinas of mice expressing the mutant human *ELOVL4* transgene did not affect the rate of retinal degeneration [34].

DHA cannot be synthesized de novo by animals and it, or its short-chain PUFA precursors, must be furnished in the diet. The *fat-1* gene, cloned from *Caenorhabditis elegans* [35], encodes an n3 fatty acid desaturase that can convert n6 to n3 PUFA. Mice expressing the *fat-1* transgene, reared on a diet containing only n6 PUFA, have significantly higher levels of DHA and other n3 PUFA compared to wild-type controls in all tissues examined, including the retina [34].

Mice expressing an opsin transgene containing three (V20G, P23H, and P27L [VPP]) mutations near the N-terminus [36] have been used extensively to study inherited retinal degeneration [37–42]. The P23H mutation in the rhodopsin gene was the first identified as a cause of autosomal dominant retinitis pigmentosa in humans [43]. VPP mice have an age-related progressive photoreceptor degeneration, with a reduction in the length of the ROS and a decreased number of nuclei in the outer nuclear layer (ONL) [36,38].

The purpose of the present study was to determine whether providing high levels of DHA in mice by expressing the fat-1 protein can protect against hereditary retinal degenerations caused by the VPP rhodopsin mutation. Using the *fat-1* transgenic animals to provide different levels of retinal DHA eliminated the need to use two diets and allowed us to use littermates as controls.

## Methods

### Animals

Transgenic mice carrying the *fat-1* gene of *Caenorhabditis elegans* in the C57BL/6J background were kindly provided by Dr. Jing Kang (Department of Medicine, Massachusetts General Hospital and Harvard Medical School, Boston, MA). Heterozygous *fat-1* C57BL/6J male mice were bred to heterozygous female mice expressing the *VPP* transgene (kindly provided by Dr. Connie Cepko, Harvard University, Boston, MA) in a C57BL/6J background that, for three weeks before breeding, had been fed a safflower oil (SFO) diet [44] (Modified AIN-76A Purified Rodent Diet with 10% SFO by weight, prepared by Dyets, Inc., Bethlehem, PA). Four different groups of mice, Fat1^+^/VPP^+^, Fat1^–^/VPP^+^, Fat1^+^/VPP^–^, and Fat1^–^/VPP^–^ were produced within the same litters at the Dean McGee Eye Institute vivarium and maintained under 25–75 lux cyclic light (12 h:12 h light-dark cycle). At weaning, all pups were maintained on the SFO diet for the entirety of the study. The SFO diet had an n6/n3 ratio of 273 and was composed of 72 molar % 18:2n6 (Table 1). Animals were cared for and handled according to the Association for Research in Vision and Ophthalmology statement for the use of animals in vision and ophthalmic research. Protocols were reviewed and approved by the Institutional Animal Care and Use Committees of the University of Oklahoma Health Sciences Center and the Dean A. McGee Eye Institute.

**Table 1:** Relative mole percentage ± standard deviation (n=3–7) of fatty acids in liver phospholipids, plasma phospholipids, retina total lipids, and chow total lipids.

**Age**	**Group**	**18:2n6**	**20:4n6**	**22:4n6**	**22:5n6**	**20:5n3**	**22:6n3**	**n6/n3**
4 wk	Plasma F+/V+	23.2±3.0	8.0±0.9	0.21±0.06	0.50±0.13	0.44±0.10	3.4±0.1	6.9±1.1
	Plasma F-/V+	21.8±2.0	11.6±2.1	0.75±0.22	3.17±0.62	0	0.5±0.1	62.4±17.2
	Plasma F+/V-	23.9±1.4	8.5±1.1	0.22±0.05	0.62±0.13	0.50±0.18	4.1±1.0	6.8±1.3
	Plasma F-/V-	21.1±1.8	11.7±1.6	0.60±0.21	3.25±0.51	0	0.4±0.1	64.5±8.6
4 wk	Liver F+/V+	21.3±2.1	15.7±2.2	0.3±0.1	0.9±0.7	0.8±0.6	6.7±0.8	5.0±0.4
	Liver F-/V+	21.0±1.0	19.5±1.4	0.7±0.0	5.1±0.4	0	0.8±0.2	58.4±10.8
	Liver F+/V-	20.8±1.8	15.9±1.0	0.3±0.0	0.9±0.2	0.6±0.3	7.8±1.2	4.6±0.7
	Liver F-/V-	20.3±1.2	19.1±0.9	0.6±0.1	4.9±0.8	0	0.7±0.1	56.3±7.3
4 wk	Retina F+/V+	2.2±0.4	7.2±0.5	0.8±0.2	0.4±0.2	0.5±0.2	21.7±1.9	0.5±0.1
	Retina F-/V+	1.4±0.1	9.2±0.2	2.6±0.2	15.1±1.6	0	6.4±1.3	4.6±1.0
	Retina F+/V-	1.8±0.7	6.5±0.4	0.9±0.1	0.7±0.1	0.3±0.1	28.2±1.2	0.4±0.1
	Retina F-/V-	1.6±0.1	9.1±0.5	3.0±0.2	18.3±1.4	0	6.2±2.0	5.6±1.7
28 wk	Plasma F+/V+	23.0±4.7	10.8±2.2	0.3±0.1	0.8±0.3	0.4±0.2	4.4±0.4	6.6±0.5
	Plasma F-/V+	22.4±2.7	13.4±0.6	0.8±0.2	3.2±0.4	0	0.6±0.2	47.0±11.5
	Plasma F+/V-	23.9±1.9	11.8±0.7	0.2±0.1	0.7±0.1	0.3±0.1	5.0±0.6	6.6±1.0
	Plasma F-/V-	21.3±2.0	14.4±1.8	0.7±0.2	3.8±0.4	0	0.7±0.3	48.7±13.7
28 wk	Liver F+/V+	18.6±1.2	18.1±2.9	0.4±0.0	1.2±0.3	0.4±0.3	8.4±1.5	4.5±0.9
	Liver F-/V+	20.6±1.2	21.5±1.1	0.7±0.1	4.8±0.8	0	1.1±0.3	42.5±8.3
	Liver F+/V-	19.7±0.7	17.8±1.2	0.4±0.0	0.9±0.2	0.6±0.3	8.0±0.6	4.5±0.5
	Liver F-/V-	19.8±2.1	20.2±1.4	0.8±0.2	6.0±1.2	0	1.2±0.5	38.2±10.3
28 wk	Retina F+/V+	1.3±0.1	9.3±0.2	1.0±0.0	0.3±0.1	0.5±0.3	21.7±1.3	0.5±0.0
	Retina F-/V+	1.4±0.3	10.8±0.7	2.0±0.2	4.3±0.6	0	13.8±1.8	1.4±0.2
	Retina F+/V-	1.4±0.2	6.4±0.3	0.9±0.0	0.3±0.1	0.2±0.0	32.5±0.6	0.3±0.0
	Retina F-/V-	1.6±0.5	8.7±0.4	2.3±0.2	8.3±2.5	0	19.4±3.6	1.2±0.5
Chow	Dyets 10% SO	72.4±0.7	0	0	0	0.1±0.0	0	273.5±5.2

In the table, F indicates fat-1 and V indicates VPP.

### Genotyping

Two millimeter tail snips were digested overnight with DirectPCR tail lysis reagent (Viagen Biotech, Los Angeles, CA) containing 0.3 mg/ml of Proteinase K (Sigma, St Louis, MO) at 55 °C. The lysates were incubated at 95 °C for 5 min. Two μl of lysate were used per PCR reaction in Green Go Taq master mix (Promega, Fitchburg, WI). The *fat-1* transgene was detected using primers (see Table 2) at 0.5 μM each. The PCR product (251 bp) was visualized on a 1.25% agarose gel. A separate PCR reaction was used to detect the *VPP* transgene. Four primers were used (Table 2). The PCR products were visualized on a 1.25% agarose gel. The PCR product of 450 bp identified the *VPP* transgene, 750 bp identified actin (internal control), and 300 bp identified an actin pseudogene with varied amplification. The genetic identity of mice expressing the *fat-1* gene was confirmed by fatty acid analysis. Identity of mice expressing the VPP mutation was further confirmed by electroretinography (ERG) and quantitative histology.

**Table 2:** The following primers were used to identify the *fat-1* transgene and *VPP* transgene.

**Gene**	**Forward primer (5′-3′)**	**Reverse primer (5′-3′)**
Fat-1	CTGCACCACGCCTTCACCACCC	ACACAGCAGATTCCAGAGATT
VPP	CAGCTGCTCGAAGTGACTCCGACC	AGACTGACATGGGGAGGAATTCCCAGA
VPP	GACAACGGCTCTGGCCTGGTG	GTGTGGCAGGGCATAGCCCTC

### Electroretinography

Animals were kept in total darkness overnight and prepared for the ERG study under dim red light (Espion E^2^ ERG System, Diagnosys LLC, Lowell, MA). Mice were anesthetized with ketamine (85 mg/kg bodyweight intraperitoneal) and xylazine (14 mg/kg bodyweight intraperitoneal). One drop of 10% phenylephrine was applied to the cornea to dilate the pupil and one drop of 0.5% proparacaine HCl was applied for local anesthesia. A reference electrode was positioned at the mouth and a ground electrode on the tail. Nine different flash intensities ranging from 0.001 to 2000 cd.s/m^2^ were used and ERG responses from both eyes were recorded with a gold electrode placed on the cornea. The a-wave and b-wave amplitudes from each eye were averaged and used for comparison of retinal function.

### Outer nuclear layer thickness measurement

Animals were killed by carbon dioxide asphyxiation following ERG testing. The right eye was enucleated and the superior margin marked with a dye. The eye was then fixed with Perfix (20% isopropanol, 2% trichloroacetic acid, 4% paraformaldehyde, and 2% zinc chloride) and embedded in paraffin, and 5 µm thick sections were taken along the vertical meridian to allow comparison of all regions of the retina in the superior and inferior hemispheres. In each hemisphere, ONL thickness was measured in nine defined areas, starting at the optic nerve head and extending along the vertical meridian toward the superior and inferior ora serrata. Measurements were made at 225 µm intervals. The mean ONL thickness was calculated for the entire retinal section, as was the ONL thickness of the superior region of retina, which is most sensitive to the damaging effects of light.

### Lipid analysis

Fatty acid profiles were analyzed in the whole retina total lipids, plasma phospholipids, and liver phospholipids from Fat1^+^/VPP^+^, Fat1^–^/VPP^+^, Fat1^+^/VPP^–^, and Fat1^–^/VPP^–^ mice by capillary column gas chromatography with a flame ionization detector [44]. Each analysis was of a single retina from four to six different mice from each group. Blood was taken by heart puncture after death and each 100 µl plasma sample was obtained by centrifuging the heparinized blood at 2,000× g in ethylene glycol tetraacetic acid-containing tubes. Retina and plasma samples were extracted by a modified Bligh and Dyer procedure, liver samples were extracted by a modified Folch procedure, and their fatty acids (as methyl esters) were analyzed using an Agilent Technologies 6890 gas chromatograph with flame ionization detector [44].

### Rhodopsin assay

Rhodopsin assays were performed as previously described [44]. Briefly, mice were dark-adapted overnight and killed under dim red light by carbon dioxide asphyxiation. Single eyes were removed and homogenized in buffer containing 10 mM Tris (pH 7.4), 150 mM NaCl, 1 mM EDTA, 2% (wt/vol) octylglucoside, and 50 μM hydroxylamine. Homogenates maintained in dim red light were centrifuged at 16,000× g and the soluble fractions were scanned from 270 to 800 nm in an Ultrospec 3000 UV/Vis spectrophotometer (GE Healthcare, Piscataway, NJ). The samples were subsequently bleached by exposure to room light (~15 min) and scanned again. The difference in absorbance at 500 nm between pre- and postbleached samples was used to determine rhodopsin content using a molar extinction coefficient of 42,000 M^–1^.

### Statistical analysis

Results are expressed as the mean±standard deviation. Differences were assessed by the Student *t* test. A value of p<0.05 was considered significant.

## Results

### Fatty acid composition

**Figure 1:**
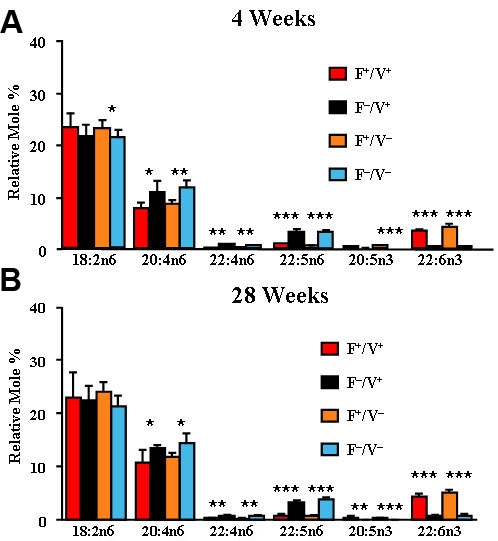
Plasma fatty acid composition. **A**: The relative mole percentage of n3 and n6 polyunsaturated fatty acids from total phospholipid extracts of plasma of Fat1+/VPP+, Fat1–/VPP+, Fat1+/VPP–, and Fat1–/VPP– mice are shown at 4 weeks of age. **B**: Relative mole percentages at 28 weeks of age are shown. (n=3–7) *p<0.05 *fat-1* positive versus *fat-1* negative. **p<0.01 *fat-1* positive versus *fat-1* negative. ***p<0.001 *fat-1* positive versus *fat-1* negative. F indicates *fat-1* and V indicates VPP.

**Figure 2:**
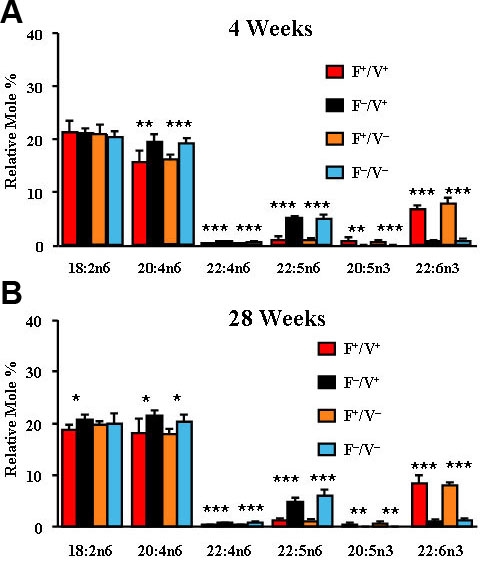
Liver fatty acid composition. **A**: The relative mole percentage of n3 and n6 polyunsaturated fatty acids from total phospholipid extracts of liver of Fat1+/VPP+, Fat1–/VPP+, Fat1+/VPP–, and Fat1–/VPP– mice are shown at 4 weeks of age. **B**: Relative mole percentages at 28 weeks of age are shown. (n=4–7) *p<0.05 *fat-1* positive versus *fat-1* negative. **p<0.01 *fat-1* positive versus fat-1 negative. ***p<0.001 *fat-1* positive versus *fat-1* negative.

We analyzed the fatty acid composition of retina total lipids, plasma phospholipids, and liver phospholipids from the four groups of mice at 4 and 28 weeks of age (see Table 1) for levels of polyunsaturated fatty acids. A complete fatty acid analysis of similar animals is given in the Supplemental Material [44]. Fatty acid composition of plasma (Figure 1; n=3–7) and liver (Figure 2; n=4–7) show significantly higher levels of n3 (p<0.01–0.001) and lower levels of n6 PUFA (p<0.05–0.001) in the *fat-1* mice at 4 weeks (Figure 1A, 2A) and 28 weeks (Figure 1B, Figure 2B) of age, compared to their *fat-1* negative littermates. Consequently, the n6/n3 ratios for *fat-1* mice were significantly lower than their *fat-1* negative littermates (Table 1). There were only minor differences in the levels of 18:2n6, the sole source of PUFA in the diet. The expression of the transgene had no effect on saturated and monoenoic fatty acid levels in the plasma or liver.

**Figure 3:**
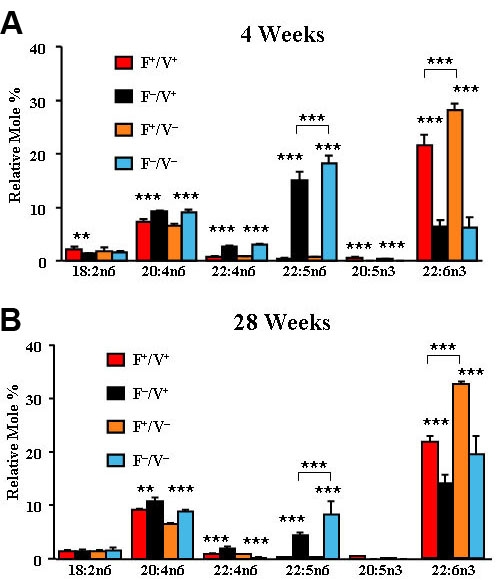
Retina fatty acid compositions. **A**: The relative mole percentage of n3 and n6 polyunsaturated fatty acids from total lipid extracts of retina of Fat1+/VPP+, Fat1–/VPP+, Fat1+/VPP–, and Fat1–/VPP– mice are shown at 4 weeks of age. **B**: Relative mole percentages at 28 weeks of age are shown. (n=4–6) **p<0.01 *fat-1* positive versus *fat-1* negative. ***p<0.001 *fat-1* positive versus *fat-1* negative.

The high levels of 18:2n6 in plasma and liver were not found in the retina (Figure 3), where the major fatty acids were C-20 and C-22 PUFA (n=4–6). At 4 (Figure 3A) and 28 (Figure 3B) weeks of age, the *fat-1* mice in both groups had significantly higher percentages of 20:5n3 (p<0.01–0.001) and 22:6n3 (p<0.001), and significantly lower percentages of 20:4n6 (p<0.01–0.001), 22:4n6 (p<0.001), and 22:5n6 (p<0.001), compared to the *fat-1* negative mice (Table 1). All four groups had a significantly higher percentage of 22:6n3 (p<0.001) and lower percentage of 22:5n6 (p<0.001) at the age of 28 weeks than at 4 weeks (Table 1). With the exception of 22:6n3 in the four-week-old retinas, there was significantly less 22:5n6 and 22:6n3 in the mice expressing the *VPP* transgene, a finding we have obtained in other mouse [27,28] and rat [29,33] retinas that express a mutant gene involved in retinal degeneration.

### Retinal structure

**Figure 4:**
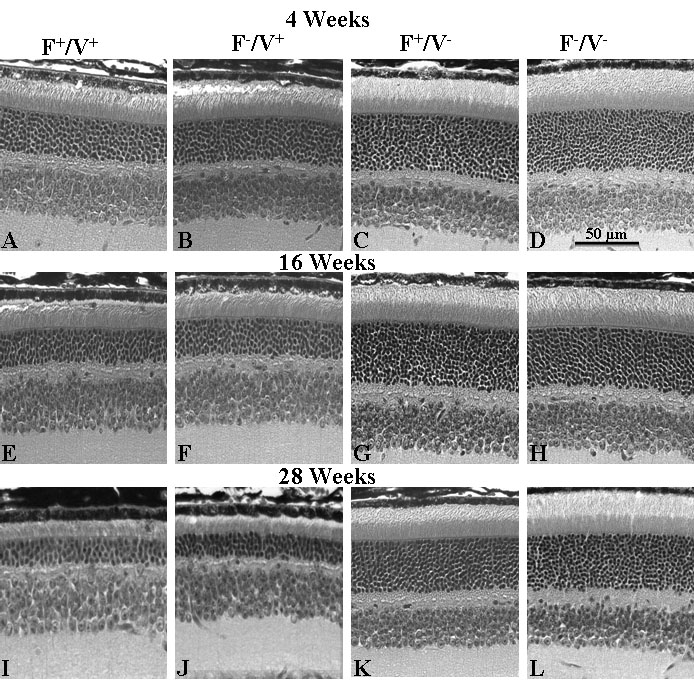
Retinal structure. **A**-**D**: Retinal structure is revealed by representative photomicrographs from the central superior retina from Fat1+/VPP+, Fat1–/VPP+, Fat1+/VPP–, and Fat1–/VPP– mice at 4 weeks of age. **E**-**H**: Photomicrographs at 16 weeks of age are shown. **I**-**L**: Photomicrographs at 28 weeks of age are shown. Magnification bar equal to 50 microns.

Retinal structure was examined along the vertical meridian from inferior to superior ora serrata. Sections from central superior retina are shown in Figure 4. At four weeks of age, the ONL thickness in both VPP^+^ groups was already reduced from the normal 10–11 rows to 6–8 rows (Figure 4A,B). By 16 weeks, more than half of photoreceptor nuclei were lost in VPP^+^ groups (Figure 4E,F). Even greater retinal degeneration is evident in the 28-week-old VPP^+^ mice (Figure 4I,J). There was some age-related loss of rod nuclei in the VPP^-^ mice (Figure 4C,D,G,H,K,L), but not to the extent seen in the VPP^+^ mice. Examination of the sections did not show any effect of 22:6n3 level on the rate of retinal degeneration in the VPP^+^ mice.

**Figure 5:**
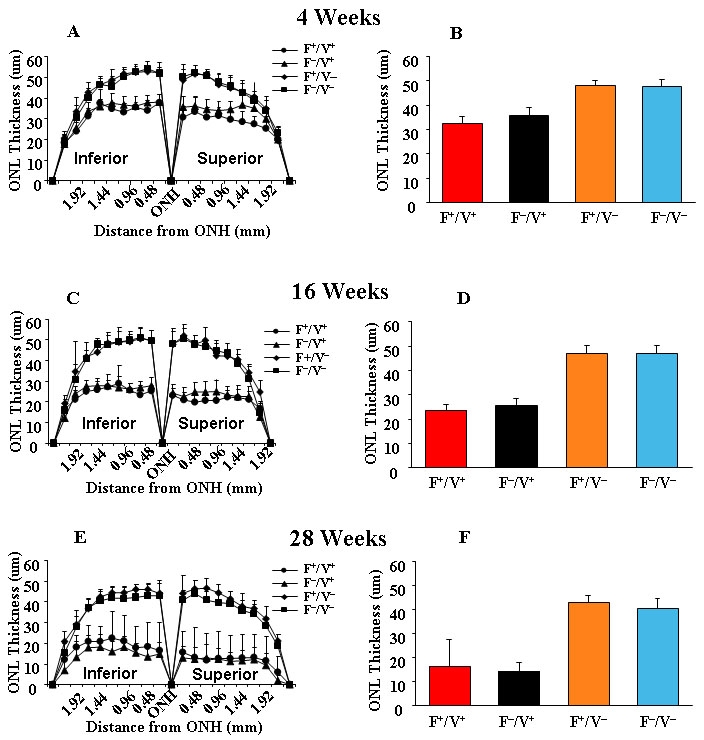
Quantification of morphological changes. The quantification of morphological changes are shown by measurements of outer nuclear layer (ONL) thickness along the vertical meridian of the eye in Fat1+/VPP+, Fat1–/VPP+, Fat1+/VPP–, and Fat1–/VPP– mice maintained in 20 lx cyclic light (n=10). **A**, **C**, **E**: Plots show ONL thickness of transgenic mice at 4, 16, and 28 weeks of age. **B**, **D**, **F**: Histograms show the average ONL thickness in the three age groups.

The progression of retinal degeneration along the vertical meridian with increasing age was quantified in the “spider” graph in Figure 5A,C,E (n=10). There was a significant reduction in ONL thickness in both inferior and superior regions of both groups of four-week-old VPP^+^ mice (Figure 5A). Means of ONL thickness taken from 14 sites along the vertical meridian (seven superior and seven inferior of the optic nerve head) were averaged for each age group. There were no significant differences in ONL thickness between Fat1^+^/VPP^+^ and Fat1^–^/VPP^+^ or between Fat1^+^/VPP^–^ and Fat1^–^/VPP^–^ mice at the three ages examined (Figure 5B,D,E). There was an age-related loss of rod nuclei in all groups, which was significantly greater in the VPP^+^ mice. However, there was no significant effect of retinal 22:6n3 levels on the rate of rod photoreceptor degeneration.

### Retinal function

**Figure 6:**
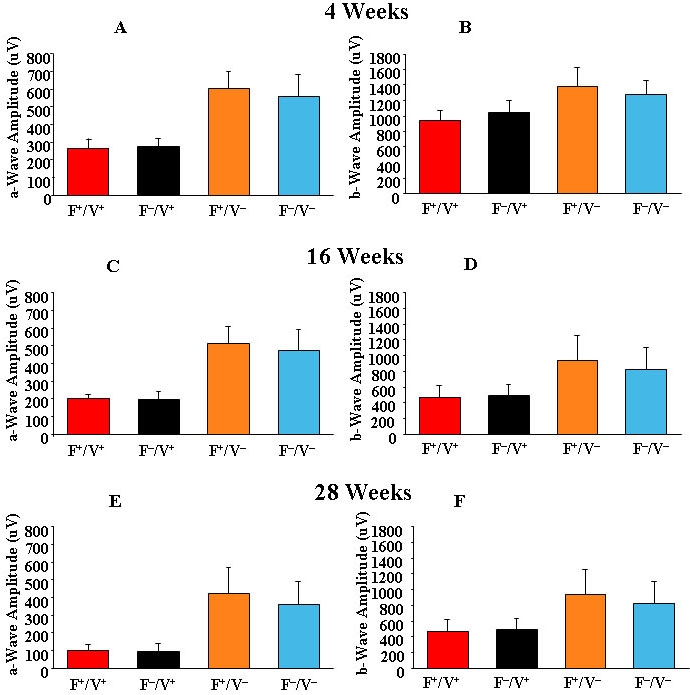
Evaluation of retinal function by electroretinography. Evaluation of retinal function by electroretinography was determined by taking measurements of a- and b-wave amplitudes in Fat1+/VPP+, Fat1–/VPP+, Fat1+/VPP–, and Fat1–/VPP– mice (n=10). **A**, **C**, **E**: The average a-wave amplitudes are shown at 4, 16, and 28 weeks of age, respectively. **B**, **D**, **F**: The corresponding b-wave amplitudes are shown.

Retinal function was determined by ERG. Nine intensities were used and the responses from both eyes were recorded simultaneously and averaged. The a- and b-wave amplitudes at a saturating flash intensity of 1,000 cd.s/m^2^ are shown in Figure 6 (n=10). There was a significant reduction in amplitudes with the age, with VPP^+^ mice showing the greatest change. Compared to four-week-old mice (Figure 6A), the a-wave amplitude in 16-week-old VPP^+^ mice was reduced by 24%–29% (Figure 6C) and by greater than 60% in 28-week-old mice (Figure 6E). The a-wave was also reduced in VPP^–^ mice by 15%–17% at 16 weeks and 29%–36% at 28 weeks. The loss of a-wave amplitude was greater in the VPP^+^ mice than in the VPP^–^ mice. There was no effect of retinal 22:6n3 levels on the a-wave amplitude in VPP^+^ and VPP^–^ mice.

Among *VPP*^+^ transgenic mice, b-wave amplitudes were reduced by 17%–29% at 16 weeks of age (Figure 6D) and by over 50% at 28 weeks of age (Figure 6F). In VPP^–^ mice, the b-wave amplitudes were reduced by 18%–19% at 16 weeks (Figure 6D) and 32%–35% at 28 weeks (Figure 6F). There were no significant differences in b-wave amplitudes between Fat1^+^/VPP^+^ and Fat1^–^/VPP^+^ or between Fat1^+^/VPP^–^ and Fat1^–^/VPP^–^ mice at any age.

### Rhodopsin levels

**Figure 7:**
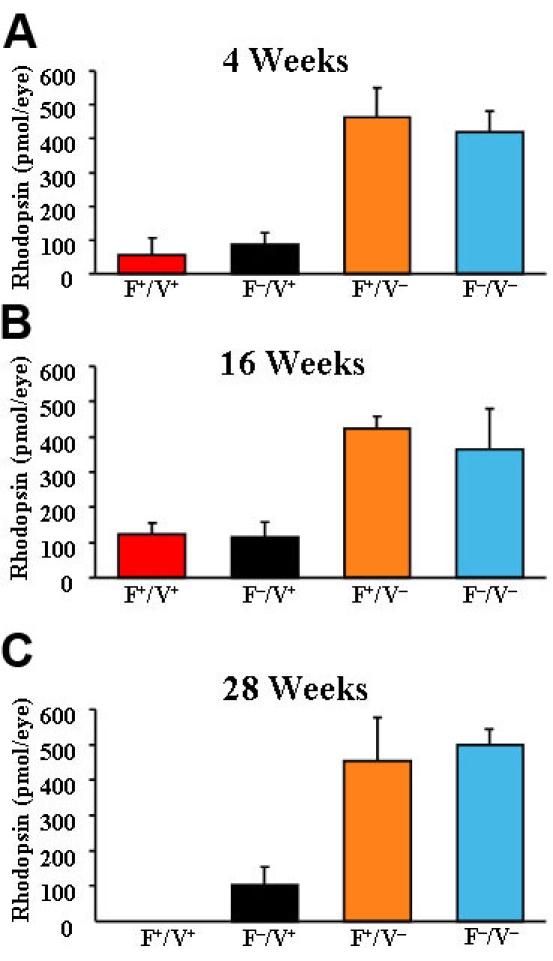
Rhodopsin content of transgenic mouse retinas. **A**: Rhodopsin content of eyes from Fat1+/VPP+, Fat1–/VPP+, Fat1+/VPP–, and Fat1–/VPP– mice was determined at age of 4 weeks (n≥6). **B**: Rhodopsin content shown at 16 weeks (n≥4). **C**: Rhodopsin content shown at 28 weeks (n≥3).

Figure 7 shows the retinal content of bleachable rhodopsin in detergent extracts of whole mouse eyes at age of four weeks (Figure 7A, n≥6), 16 weeks (Figure 7B, n≥4), and 28 weeks (Figure 7C, n≥3). *VPP* transgenic mice had low amounts of rhodopsin. There were no significant differences between Fat1^+^/VPP^+^ and Fat1^–^/VPP^+^ or between Fat1^+^/VPP^–^ and Fat1^–^/VPP^–^ mice at any of the three age groups. Statistical analysis was not possible at 28 weeks of age as no rhodopsin was detectable in Fat1^+^/VPP^+^ mice at this age.

## Discussion

In 1993, Naash and colleagues [36] generated a transgenic mouse with three point mutations in rhodopsin (V20G, P23H, and P27L; termed VPP) that resembles the P23H mutation found in many humans with autosomal dominant retinitis pigmentosa [43]. VPP mice have reduced levels of rhodopsin and amplitudes of a- and b-waves of their scotopic ERG compared to nontransgenic littermates [36]. They also have structural abnormalities, including shortened rod outer segments and progressive loss of rod nuclei [36]. The VPP mice used in the present study displayed all of these characteristics.

Over the last three decades, studies by many laboratories [11–13,15–19,21], including our own [14,20], have shown that patients with retinitis pigmentosa and Usher’s disease have lower levels of 22:6n3 and other PUFA in plasma, serum, and red blood cells. The cause of the reduced PUFA is not known, but it does not appear to be related to diet, exercise, or type of inheritance. We have reported that the levels of DHA are lower in the retinas of dogs [25,26] and in rod outer segments of mice [27,28] and rats [29] that express transgenes of naturally occurring mutations that cause retinal degenerations in humans. However, attempts to slow the degeneration in dogs with progressive rod-cone degeneration by providing fish oil were not successful [25]. Likewise, when rats expressing the P23H or S334ter rhodopsin transgenes were raised on the SFO diet, which reduced their ROS DHA by 50%, there was no exacerbation of rod photoreceptor cell loss [29].

Mammalian species cannot naturally produce n3 from n6 PUFA and must therefore depend on a dietary source for these essential fatty acids. The *fat-1* transgenic mouse expresses an n3 fatty acid desaturase, derived from *C. elegans* [35], that can convert n6 to n3 PUFA by introducing a double bond into n6 PUFA at the n3 position of their hydrocarbon chains [45,46]. This genetic manipulation allows significant fatty acid compositional changes to be made in cellular lipids in mice fed the same diet and born to the same group of dams. The SFO diet, which contains mainly 18:2n6 and only traces of n3 PUFA, has been used in many studies [2,44,47–49] to reduce n3 PUFA in membrane phospholipids. All of the mice used in the present study were fed the SFO diet, with the dams started several weeks before breeding. This ensured that, during development and the nursing period following birth, the pups were supplied primarily with n6 PUFA, since fatty acids in the blood and the milk of the dam reflect the fatty acid composition of their diet.

In the present study, we used genetic manipulation rather than diet to alter the DHA content of the retina of a mouse model of human RP. Our results show a significantly higher level of DHA (p<0.001) in the *fat-1* positive mice compared to the *fat-1* negative mice in the retina, plasma, and liver. The retina had the highest levels of DHA, which were comparable to those reported previously for *fat-1* positive mice [34,44] and for mice [27,28] and rats [33,49] fed diets containing n3 PUFA. Retinas of *fat-1* negative mice fed the SFO diet had about half the DHA levels of the *fat-1* positive mice, but these reduced DHA levels did not result in changes in retinal structure or function as we recently reported [44]. In this earlier study, dietary n3 deficiency in *fat-1* negative animals resulted in only subtle electroretinographic changes (e.g., decreased a-wave sensitivity and increased a-wave implicit times), but did not result in profound changes in ERG amplitudes or retinal structure unless stressed by intense light exposure [44].

The level of DHA in the retinas of VPP mice had no influence on the rate of loss of photoreceptor cells. There were no functional or structural differences between the *fat-1* positive and negative mice either in the presence or absence of the *VPP* transgene. Thus, the presence of a twofold higher content of DHA in the retinas of mice expressing a mutation causing human RP could not rescue the rod photoreceptor cells from death. We reached a similar conclusion in a recent study in which mice expressing a transgene of human mutant *ELOVL4* gene were bred with *fat-1* mice to generate animals with high and low levels of retinal DHA [34]. A DHA-derived docosanoid product, neuroprotectin D1, has potent neuroprotective effects through the suppression of inflammation (reviewed in [50]). The lack of protective effect of increased DHA levels on the progression of degeneration in the VPP mouse suggests the possibility that inflammation does not contribute significantly to the pathogenesis of this particular model. Therefore, on the basis of several studies showing no beneficial effects of DHA on the survival of retinal rod photoreceptors in mice expressing mutant genes or mutant transgenes, we conclude that DHA does not rescue these cells from degeneration.
